# Nitrogen waste metabolism as a locus of nitrergic co-transmission in the brain

**DOI:** 10.3389/fnbeh.2025.1537975

**Published:** 2025-10-06

**Authors:** Joseph L. Bedont

**Affiliations:** Department of Biological Sciences, Kent State University, Kent, OH, United States

**Keywords:** nitric oxide, polyamines, polyamine-derived GABA, metabolism, behavior, sleep, addiction, learning

## Abstract

Nitrogen detoxification pathways in the central nervous system supply a range of neurotransmitters, ranging from long-appreciated examples like nitric oxide and agmatine, to emergent neurotransmitters including spermidine, spermine, and polyamine-derived GABA. This review summarizes specialized nitrogen detoxification pathways in the brain, and evidence supporting several of these pathways’ metabolites as co-transmitters in neurons and glia. Known functional roles of these nitrergic co-transmitters in learning, sleep, addiction, and other neurological disorders will be discussed to elucidate the adaptive value of nitrergic co-transmission, with a particular focus on nitrergic modulation of coincidence detection at NMDA receptors. Finally, this review sums up with a discussion of how nitrogen homeostasis in the brain serves as a coordinating locus for the control of these nitrergic neurotransmitters, and approaches for identifying *bona fide* co-transmitter effects of these metabolites in future work.

## Introduction

It is well-known that the brain is susceptible to oxidative stress, for reasons including high metabolic needs, oxidative stress associated with the production and storage of neurotransmitters, and sparse antioxidant defenses (Cobley et al., 2018). Less frequently appreciated is that the brain is highly susceptible to nitrogen stress, with ammonia elevation in particular having devastating consequences (Bosoi and Rose, 2009; Jo et al., 2021). Indeed, accumulating nitrogen stress may drive many negative outcomes of aging in the brain, including neurodegeneration.

To combat this, the brain has evolved modified nitrogen metabolism. The urea cycle is largely acyclical under physiological conditions in the brain, reducing the amount of nitrogen stress that is locally converted to urea. Instead, excess nitrogen is largely transiently stored as glutamine, primarily in astrocytes that are less sensitive to nitrogen stress than neurons (Cooper, 2001; Bak et al., 2006; Cooper and Jeitner, 2016). This “buffered” nitrogen stress is then exported to the periphery over time, where it makes its way to the liver, kidneys, and other organs. With their complete urea cycle activity, lower sensitivity than the brain to nitrogen stress, and ultimately access to excretion capacity to remove excess nitrogen from the body, these peripheral organs carry out the heavy lifting of eliminating nitrogen stress from the body entirely (Lee et al., 1998; Cooper and Jeitner, 2016).

Thus, while the primary focus of this review is on the repurposing of nitrergic metabolic pathways in the brain into co-transmission networks, it is important to acknowledge that the overall tone of these networks’ activity often begins and ends in crosstalk with the rest of the body. The exchange of nitrogen equivalents with the periphery heavily influences nitrogen load in the brain, and in turn both absolute levels and ratios of nitrergic co-transmission. In part, this reflects excitation/inhibition balance and classical aminergic neuromodulatory tone in the brain, both of which are deranged by excess nitrogen and can ultimately cause excitotoxicity if nitrogen imbalances are not promptly addressed (Albrecht, 2007; Rangroo Thrane et al., 2013). More directly, nitrogen load also directly impacts the equilibrium of nitrogen equivalents between astrocytic glutamine storage and other nitrogen-rich metabolites including L-arginine, which is a key substrate for synthesizing many nitrergic metabolites (Ligthart-Melis et al., 2008; Morris et al., 2017).

This arginine-derived metabolic network includes the ureotelic nitrogen metabolites that are the primary focus of this review article: nitric oxide, polyamines, and their derivatives like polyamine-derived GABA. These molecules have been co-opted by the brain to function as neurotransmitters, often alongside more traditional neurotransmitters as metabolism-sensitive co-transmitters. By examining how these nitrergic co-transmitters exert key influences on physiological functions including learning and sleep in this review, I will seek connections to their known involvement in pathological states including addiction, psychiatric illness, and neurodegeneration. Ultimately, I will seek to integrate this information into a coherent hypothesis of how nitrergic co-transmission coupled to the brain’s nitrogen load may function in rationally coordinated ways to help maintain nitrogen homeostasis. I then wrap up by discussing some promising approaches to expand our understanding of this network going forward. To aid in quickly referencing the somewhat large number of abbreviations used in addressing these broad themes, readers may refer to Table 1.

**TABLE 1:** Glossary of frequently used acronyms and abbreviations.

Abbreviation	Full name	Definition/functions relevant to this review article
AD	Alzheimer’s disease	A prevalent form of dementia resulting from amyloid and tau protein pathology, with many links to nitrergic co-transmitters
Agm	Agmatine	Polyamine with the longest track record as a co-transmitter, especially in Glu/Agm neurons, including established roles in plasticity, addiction, and other psychiatric disease
Aldh1a1	Aldehyde dehydrogenase 1a1	Enzyme involved in synthesis of GABA from polyamines
AMPA	a subclass of glutamate receptor	A more directly faithful Glu receptor that primarily fluxes depolarizing sodium, but not calcium
DA	Dopamine or dopaminergic	Classical monoamine neurotransmitter involved in reward, addiction, and wake promotion
eNOS	Endothelial nitric oxide synthase	NOS isoform enriched in brain vasculature
GAD	Glutamate decarboxylase	Enzyme that synthesizes GABA by decarboxylating Glu
GABA	Gamma-amino butyric acid	The mammalian brain’s primary inhibitory neurotransmitter
GAT1	GABA transporter 1	Re-uptakes released GABA from the synaptic cleft
Glu	L-glutamate	The mammalian brain’s primary excitatory neurotransmitter
iNOS	Inducible nitric oxide synthase	NOS isoform enriched in glia, often associated with neuroinflammation
IPSC	Inhibitory post-synaptic current	Neurotransmission (often GABA) driven inhibitory currents in receptive neurons
NMDA	A subclass of glutamate receptor	A coincidence detecting receptor that fluxes plasticity-inducing calcium, the opening of which is heavily regulated by Glu, membrane depolarization, and many nitrergic co-transmitters
nNOS	Neuronal nitric oxide synthase	NOS isoform enriched in neurons, often associated with neuroprotection
NO	Nitric oxide	Gaseous retrograde and anterograde neurotransmitter widely involved in plasticity, sleep, reward, and brain pathology, often downstream of NMDA activation
NOS	Nitric oxide synthase	Enzyme that produces NO from the decomposition of L-arginine into citrulline
Put	Putrescine	While not yet shown to itself be a neurotransmitter, this metabolite is a source of Spd, Spm, and oxidatively-synthesized GABA
Spd/Spm	Spermidine/spermine	These polyamines’ roles as co-transmitters have only relatively recently been appreciated, including a likely role in learning and memory
VMAT2	Vesicular monoamine transporter 2	A transporter canonically involved in vesicular loading of monoamine neurotransmitters, but also likely loads GABA co-transmitter in some monoaminergic neuron populations
VPAT	Vesicular polyamine transporter	A relatively recently discovered transporter that is able to load a number of polyamine and canonical monoamine neurotransmitters for vesicular release

## Nitric oxide

### Nitric oxide (NO) synthesis

The best known and most thoroughly studied nitrergic co-transmitter is the gaseous neurotransmitter NO. Because of the non-vesicular nature of its release and spread as a gas, neuronal NO is generally released as a locally-active co-transmitter alongside whatever vesicular neurotransmitter(s) are released by a given neuron. Because this general schema of NO function is long established and broadly accepted, I will focus less on NO’s status as a *bona fide* co-transmitter than for other metabolites discussed in this review.

Nitric oxide synthase (NOS) is the well-conserved synthetic enzyme for NO from bacteria to mammals, but homolog duplication and deletion events have happened several times in evolutionary history (Locascio et al., 2023). For example, the fruit fly *D. melanogaster* has only one copy that can be found in several cell compartments, while mammals have 3 distinct NOS genes summarized below.

1. nNOS: a neuronal cytosolic variant commonly localized to post-synaptic densities, and generally accepted to be the most involved enzyme in physiological co-transmission
2. iNOS: a cytosolic variant expressed in a range of cells including transmission-relevant astrocytes and microglia, from which it likely plays an indirect co-transmission role
3. eNOS: a primarily endothelial membrane-localized variant that is likely the least relevant for co-transmission

**FIGURE 1:**
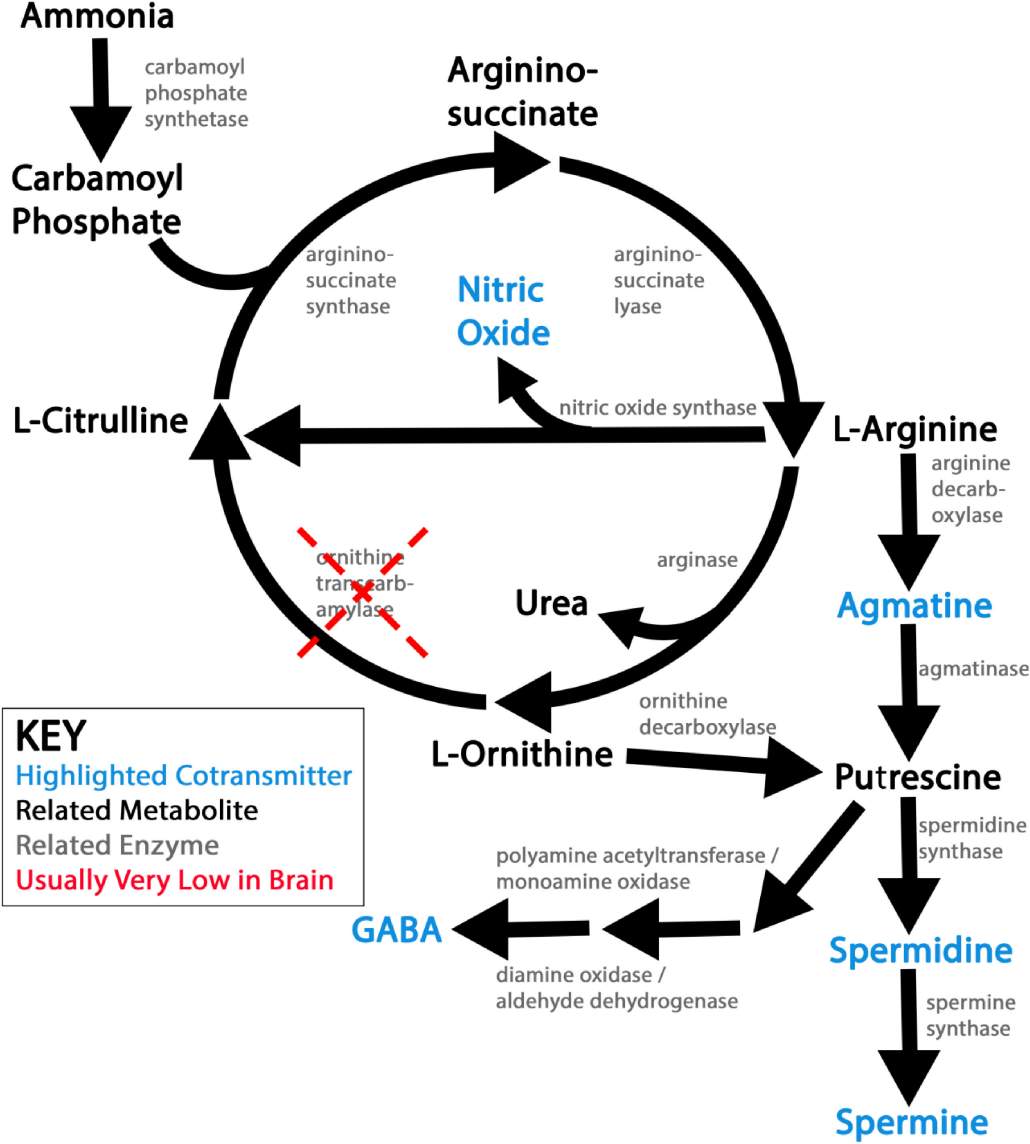
Schematic of ureotelic nitrogen waste metabolism and related nitrergic co-transmitters. This figure shows a simplified view of ureotelic nitrogen metabolism in the brain under physiological conditions. Nitrergic co-transmitters explored in this review article are flagged in blue. Note that low activity of ornithine transcarbamylase (OTC) leaves the urea pathway largely linear and non-catalytic in the brain under physiological conditions, which likely promotes the synthesis of the nitrergic co-transmitters produced by this metabolic pathway.

NOS synthesizes NO by deiminating L-arginine into L-citrulline, short circuiting the urea cycle…when it is present. However, as previously mentioned, the urea cycle is largely linear in the brain under physiological conditions, due to very low ornithine transcarbamylase activity (Figure 1; Bensemain et al., 2009; Ju et al., 2022). Since L-arginine availability is often rate-limiting for NO synthesis and subject to competition with arginase for substrate (Boucher et al., 1999; Que et al., 2002; Mori and Gotoh, 2004), linearity of urea production very likely amplifies NO co-transmission (Figure 1).

### Nitric oxide signal transduction

Transduction of NO signaling generally occurs through the oxidative modification of specific sites on target proteins, lipids, or cofactors. Its classical effector is guanylyl cyclase, which becomes activated when NO modifies its heme cofactor (Denninger and Marletta, 1999). This accelerates the enzyme’s catalytic conversion of GTP into cGMP, which can in turn modify the activity of various cGMP-sensitive enzymes, ion channels, and other effectors. NO can also reversibly nitrosylate cysteine residues and inhibit mitochondrial cytochrome c, leading to increased production of superoxide that locally reacts with NO to form peroxynitrite (the extremely reactive nucleophile ONOO-) (Cobb and Cole, 2015). Highly reactive peroxynitrite and its derivatives can then react with tyrosine residues on proteins, and fatty acids to produce often anti-inflammatory lipids (Moncada and Bolaños, 2006; Cobb and Cole, 2015; Bartesaghi and Radi, 2018). The addition of a negative charge to the normally neutral hydroxyl moiety of tyrosine can profoundly alter protein shape and function through electrostatic interactions, and nitration is often quite spatially specific due to the high reactivity of superoxide and peroxynitrite. Nitration thus couples the functions of target macromolecules to local cellular redox state.

**FIGURE 2:**
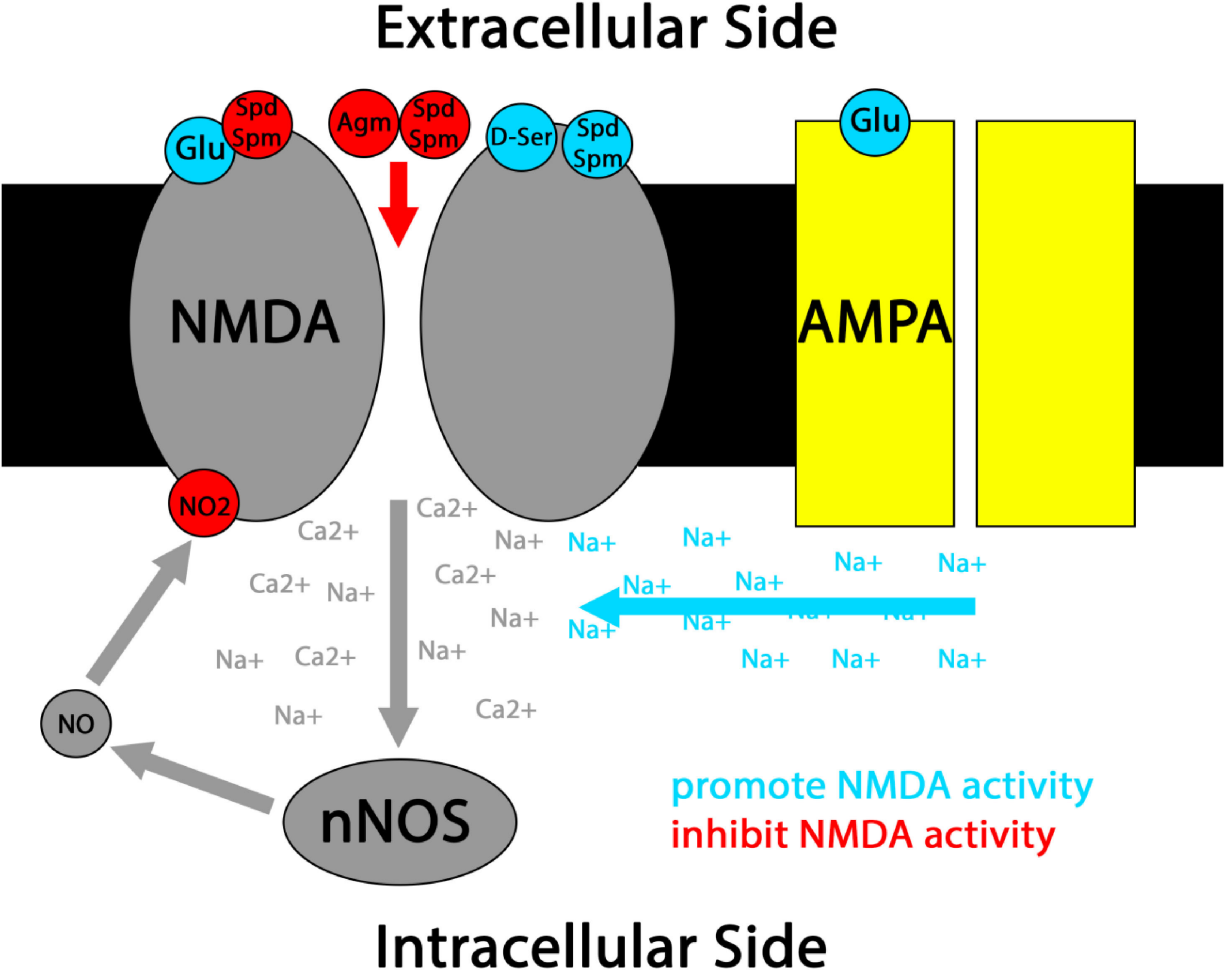
Simplified schematic of nitrergic co-transmitter interactions with classical NMDA receptor coincidence detection. Canonically, NMDA receptors open when Glu ligand binding coincides with cell membrane depolarization driven by sodium (Na+) flux through AMPA receptors and/or the binding of co-transmitters such as D-ser to NMDA receptors. When NMDA receptors open, their mixed Na+/Ca2+ flux activates effectors including nNOS. Resulting NO co-transmission can lead to nitrosylation and nitration of the NMDA receptor, generally inhibiting its further activation. Co-transmission by the polyamines Spd and Spm can have mixed effects at NMDA receptors, including NMDA activation and inhibition of Glu ligand binding. Polyamine co-transmission by Spd, Spm, and Agm have also been reported to directly block the NMDA cation channel under some conditions.

The NMDA glutamate receptor is both a major source of NOS activation and a major target of NO-driven protein nitration. NMDA receptors open their cation channels only selectively even when their primary glutamate (Glu) ligand is bound, in a manner gated by both membrane depolarization and a number of co-transmitters, including several nitrergic co-transmitters that will be revisited throughout this review (Figure 2). When open, NMDA channels flux not only sodium, but also calcium, activating a host of downstream effectors that in many neurons includes nNOS (Gunasekar et al., 1995). NMDA receptors’ requirement for coincidence detection to activate and ability to flux calcium ions makes the NMDA receptor integral to synaptic plasticity, and NO co-transmission is intimately coupled to NMDA receptor activity in this context (Hansen et al., 2017). NO mediated nitration and nitrosylation are generally thought to serve as brakes on NMDA receptor activity under physiological conditions, providing homeostatic feedback that inhibits excitotoxicity (Figure 2; Manzoni et al., 1992; Murphy et al., 1994; Murphy and Bliss, 1999; Choi et al., 2000; Chen et al., 2017; Sharma et al., 2023). While some older studies supporting this point suffer from UV artifacts related to photolytic NO uncaging (Murphy et al., 1994; Murphy and Bliss, 1999; Hopper et al., 2004), several other lines of evidence support an inhibitory role for NO-mediated modifications at the NMDA receptor (Manzoni et al., 1992; Choi et al., 2000; Chen et al., 2017; Sharma et al., 2023). That said, the polarity of NO’s effects may be reversible in some pathological contexts, including under conditions of hypoxia (Zanelli et al., 2002).

### Nitric oxide in glutamatergic LTP, learning, and memory in the hippocampus

The classical induction stimulus for NO synthesis in neurons is internal calcium elevation, often immediately downstream of NMDA receptor activation. Since NOS is often NMDA-activated in dendrites near the post-synaptic density, gaseous NO can often signal both pre- and post-synaptically at the afferent synapse. Given this localization, it is not surprising that a major physiological function of NO co-transmission is to modulate long-term potentiation (LTP) and long-term depression (LTD) of synapse strength, as a substrate to encode learning and memory.

Post-synaptically, a major mechanism for LTP is recruiting additional excitatory AMPA Glu receptors to sites of sustained NMDA receptor activation. Anterograde NO signaling promotes insertion of AMPA subunits at synapses undergoing LTP via guanylyl cyclase dependent protein kinase G (PKG) phosphorylation of its tail domain, while direct nitrosylation of multiple AMPA subunits and associated interaction partners also tend to have net LTP-promoting effects (Ivanova et al., 2020). NO-driven PKG phosphorylation also promotes *de novo* gene expression required for sustained LTP and long-term memory, through multiple distinct transcription networks (Lu et al., 1999; Gallo and Iadecola, 2011; Ivanova et al., 2020). Although less exquisitely well-characterized mechanistically, retrograde NO is also involved in presynaptic plasticity in many brain regions, often serving to reduce excitatory Glu or increase inhibitory GABA release from afferent axons (Yang and Calakos, 2013). Inconsistencies in early studies of NO’s role in LTP largely appear to have stemmed from species differences in NO synthesis, with C57BL/6J mice and human tissue exhibiting more robust hippocampal nNOS expression and LTP across the lifespan than Sv129 mice or rats (Blackshaw et al., 2003).

Accordingly, though not always without controversy, NO co-transmission has been implicated in many forms of learning. NO-mediated LTP has been implicated in hippocampal learning tasks, including explicit object recognition (Pitsikas, 2015) and spatial learning in Barnes maze and Morris water maze tasks (Majlessi et al., 2008). That said, some early studies—primarily in the rat model—did not observe significant effects of inhibiting NO synthesis on hippocampus dependent learning (ex: Bannerman et al., 1994; Prickaerts et al., 1998; Blokland et al., 1999). Partially, this may reflect how experimental protocols are structured, as NO synthesis appears to be more important during training than during consolidation or later recall of hippocampal-dependent learning (Mogensen et al., 1995; Noda et al., 1997; Qiang et al., 1997; Harooni et al., 2009). Wide use of the rat model for studies of hippocampus-dependent learning (Blackshaw et al., 2003) and drug-induced malaise at high systemic doses of some NOS inhibitors (Prendergast et al., 1997) may also have contributed to variability in results.

### Nitric oxide in other forms of learning and memory

Nitric oxide is also involved in several forms of fear learning that depend upon the amygdala and related brain regions (Medeiros et al., 2022). Most studies have shown that NO is required for both initial acquisition and recall of fear learning, but is less critical for fear maintenance (Sadeghi et al., 2022). Importantly, while NO largely seems to be dispensable for LTP involved in extinguishing fear memory, NO is often required when extinction must be generalized to new contexts (Sadeghi et al., 2022). Conversely, NO-mediated LTD is an important neural substrate of other kinds of learning, including visual recognition memory (Tamagnini et al., 2013) and cerebellar motor learning (Lev-Ram et al., 1995; Schweighofer and Ferriol, 2000; Ogasawara et al., 2007). More generally, a large preponderance of relevant studies find that NO is necessary for efficient learning and memory in many different neural circuits in healthy mammals (Majlessi et al., 2008; Pitsikas, 2015). The role of NO in learning and memory is also well-conserved across animals, with prominent examples in non-mammals including snails (Balaban et al., 2014), fruit flies (Jeong, 2024), and fish (Gómez et al., 2020).

### Nitric oxide in sleep

Nitric oxide also plays important roles in sleep homeostasis, functioning as a co-transmission loop coupling nitrogen metabolic state to sleep behavior, which in turn impacts nitrogen metabolism. Most work agrees that NO signaling in the brain promotes both baseline sleep, and increased homeostatic rebound sleep after acute sleep deprivation (Gautier-Sauvigné et al., 2005; Cespuglio et al., 2012). Conversely, sleep restriction promotes nitrogen stress accumulation; this is generally modest after acute deprivation, but can accumulate with sustained sleep loss (Everson et al., 1989; Xu et al., 2016; Malik et al., 2020; Bedont et al., 2023; Oishi et al., 2023). Neural NO production very likely increases relatively early on in sleep restriction. Indeed, nNOS induction has been noted with a 6 day sleep deprivation protocol in mice (Chittora et al., 2022), which may well serve to promote rebound sleep and in turn lower nitrogen load. However, as nitrogen stress builds with chronic sleep loss, polyamines known to inhibit nNOS also accumulate, and may impede NO-driven homeostatic sleep rebound (Hu et al., 1994; Xu et al., 2016; Bedont et al., 2023). To my knowledge, the efficacy of NO in driving sleep after very long periods of sleep restriction has not yet been examined.

Nitric oxide promotion of sleep may also support its important role in learning. The preponderance of evidence suggests that recent hippocampal learning is consolidated during sleep through the reinforcement of hippocampal replay (Ólafsdóttir et al., 2018). In particular, association of replay with broadly patterned electrophysiological activity patterns during NREM (deep) sleep, and elongated replay closer to the timescale of the original learning during REM (dreaming) sleep, are both generally believed to facilitate consolidation of learning (Louie and Wilson, 2001; Dickey et al., 2022). REM sleep also modulates metabolism and neurogenesis in ways that promote hippocampal learning; the former has been causally linked to REM learning consolidation in rodent models (Kim et al., 2021; Tripathi and Jha, 2022). That said, during sleep broad down-scaling of synapses (generally ones less active during the previous day) has been observed, which is believed to maintain plasticity for future learning (De Vivo et al., 2017; Diering et al., 2017; Tononi and Cirelli, 2020). There has been some inconsistency in the literature on the learning and memory promoting effects of REM sleep in particular, with some work describing paradoxical learning-promoting effects of REM sleep deprivation (Rasch et al., 2009; Jiang and Zhu, 2015). The relative degree of engagement of competing processes of replay-mediated consolidation and down-scaling mediated deconsolidation by particular experimental paradigms may explain much of this controversy.

Gaseous co-transmitters like NO are conceptually attractive candidates for involvement in these dual processes, as their diffusible nature could allow signaling across both recently activated and recently passive circuits within a given region in tandem. A mixture of NO-driven LTP and/or LTD resulting from differences in transduction mechanisms setup by activity during the preceding wake period could also account for results suggesting both beneficial and paradoxically detrimental effects of sleep on learning and memory. In this context, it is thought-provoking that injection of the NOS substrate L-arginine was recently reported to attenuate deficits in Morris water maze learning after REM sleep deprivation (Bodagh and Taati, 2023). While non-linear dose-dependence and possible NOS-independent effects of L-arginine complicate interpretation of this study, it nonetheless highlights NO as a promising candidate mechanism coupling sleep to learning.

### Nitric oxide in maladaptive “learning” associated with opioid addiction

Nitric oxide can be deleterious in maladaptive forms of learning acquired during addiction. One prominent example is the role of NO in withdrawal syndromes associated with many drugs of abuse, especially opioids. Correlates of neuroplasticity associated with opioid intoxication depend upon NO signaling (Peregud et al., 2016), and altered (mostly increased) NOS expression and NO synthesis during opioid withdrawal that correlate with behavioral symptoms occur in many addiction relevant brain regions (Leza et al., 1996; Peregud et al., 2006, 2008). Consistent with NOS plasticity playing a causal role in opioid withdrawal, pharmacological NOS inhibition also blunts a range of opioid withdrawal symptoms in rodent addiction models (Tayfun Uzbay and Oglesby, 2001; Kalamarides et al., 2024).

The literature is somewhat more equivocal on the effects of NO on the development of opioid tolerance. Inhibition of both NOS and peroxynitrite formation has generally been reported to antagonize opioid tolerance, especially but not exclusively when NOS is inhibited after the induction of opioid tolerance (Tayfun Uzbay and Oglesby, 2001; Muscoli et al., 2007; Ozdemir et al., 2011). Paradoxical findings have more frequently been observed when NOS manipulations are delivered *during* the induction of opioid tolerance. For example, accelerated tolerance with morphine + NOS inhibitor treatment and blunted tolerance with morphine + L-arginine treatment has been reported (Dambisya and Lee, 1996; Tayfun Uzbay and Oglesby, 2001). Opioids inhibit NOS (Kampa et al., 2001), and this inhibition may need to be well-correlated with other effects of opioids (perhaps at NMDA coincidence detectors?) in order for the plasticity required for tolerance to occur. In contrast, once the neural correlates of tolerance are established, opioid and non-opioid NOS inhibition likely compound, more easily surmounting enhanced NO synthesis associated with tolerance.

### Nitric oxide in other types of addiction

Nitric oxide involvement in addiction is not limited to opioids. Most studies examining the effects of ethanol co-administration with NOS inhibitors have found an enhancement of the acute intoxicating effects (Adams et al., 1994; Tayfun Uzbay and Oglesby, 2001). This suggests that, much like opioids, inhibition of NOS may contribute to ethanol’s mechanisms of action. In contrast, NOS inhibition during withdrawal largely seems to be beneficial. NOS induction in specific brainstem and midbrain nuclei correlates with ethanol withdrawal, and NOS inhibition in both regions specifically blunts withdrawal-driven anxiety (Bonassoli et al., 2013; Jiao et al., 2021; Zhao et al., 2021). Broad NOS inhibition also blunts, while NOS stimulation exacerbates, overall alcohol withdrawal behavior in rodent models (Adams et al., 1995; Lallemand, 1997; Tayfun Uzbay and Oglesby, 2001).

More generally, while NO has been implicated in mechanisms of intoxication and other aspects of addiction for specific drugs, including a likely role in hyperactivity induced by dopaminergic stimulants like cocaine and amphetamines (Tayfun Uzbay and Oglesby, 2001), NO is most consistently involved in neural mechanisms underlying withdrawal (i.e., sequelae of plastic changes in neural circuits that occur during drug-associated “learning”). NOS inhibition suppresses symptoms of withdrawal from nicotine (Adams and Cicero, 1998; Malin et al., 1998), and acupuncture intervention to alleviate nicotine withdrawal-driven anxiety is correlated with reduced hypothalamic NOS activation (Liu et al., 2019). NOS inhibition also blunts both sensitization and behavioral despair that occurs during amphetamine withdrawal (Liu et al., 2011; Haj-Mirzaian et al., 2018). Similarly, NOS inhibition reduces both neural correlates and behavioral symptoms including drug seeking during cocaine withdrawal, while having no effect upon cocaine seeking in naïve rodents (Bhargava and Kumar, 1997; Orsini et al., 2002; Rawls et al., 2006; Nasif et al., 2011). Salutary effects of NOS inhibition have also been noted during withdrawal from cannabinoids (Rawls et al., 2006) and benzodiazepines (Talarek et al., 2011). In sum, NOS inhibition specifically during withdrawal appears to have therapeutic potential across a range of preclinical animal models, drugs of abuse, and experimental paradigms.

### Nitric oxide in psychiatric disease

Nitric oxide’s role in maladaptive learning is not limited to addiction; it has also been implicated in the etiology of psychiatric disease. One prominent example is post-traumatic stress disorder (PTSD), which can be viewed as classical fear learning that maladaptively associates everyday conditioned stimuli with an extremely stressful inciting stimulus, leading to debilitating intrusion of the original stressor into daily life (Careaga et al., 2016). Likely through co-transmission associated with LTP-inducing NMDA signaling, NO plays a role in PTSD-associated synaptic plasticity (Sadeghi et al., 2022). Polymorphisms in nNOS and a related adaptor protein correlate with PTSD incidence and symptom severity (Lawford et al., 2013; Bruenig et al., 2017), and bioavailability of L-arginine required for NO synthesis is depleted by PTSD and negatively associated with symptom severity (Bersani et al., 2016). Most studies in preclinical models of anxiety also report anxiolytic effects of NOS inhibition (Guimarães et al., 1994; Volke et al., 1997; Yildiz et al., 2000; Harkin et al., 2004; Spiaccijr et al., 2008; Zhang et al., 2010; Pitsikas, 2024), including in the seat of fear learning: the amygdala (Forestiero et al., 2006). Given the prominent role of anxiety in PTSD and specific requirement for NO to generalize extinction of fear learning to novel contexts (Sadeghi et al., 2022), PTSD is a particularly promising candidate for therapeutic NOS inhibition. That said, it is important to note that a minority of studies using older, broad-spectrum NOS inhibitors have instead reported anxiogenic effects of NOS inhibition (Pitsikas, 2024). Selective nNOS inhibition has an especially favorable distribution of anxiolytic/anxiogenic results, suggesting that drugs focused on the co-transmission role of NO by inhibiting either nNOS itself or its specific interaction partners are particularly promising (Zhu et al., 2023; Pitsikas, 2024).

This jives with evidence suggesting that nNOS inhibition is a mediator of anxiolytic and mood-improving effects of medications used to treat both PTSD and other anxiety/mood disorders (Zhu et al., 2023). Selective serotonin reuptake inhibitors (SSRIs) are widely used to treat such disorders by inhibiting the reuptake transporter (SERT) that removes serotonin from the synaptic cleft, in turn increasing the gain of serotonin signaling in the brain. nNOS co-transmission inhibits both pre- and post-synaptic components of serotonergic synapses, including SERT, its behaviorally-relevant 5HT1A receptor, and serotonin neuron activity, and SSRIs disrupt many of these effects of nNOS (Chanrion et al., 2007; Zhang et al., 2010; Gartside et al., 2020; Shi et al., 2022). Other anti-depressants including ketamine may also function in part through inhibition of nNOS (Shen et al., 2022). Given this, it is unsurprising that co-administration of NOS inhibitors and SSRIs have synergistic benefits in preclinical models of anxiety and mood (Harkin et al., 2003, 2004; Sadeghi et al., 2023). More generally, potentially pathological changes to the NO nitrergic co-transmission system have been reported to correlate with a range of additional psychiatric conditions, including other anxiety disorders, depression, and bipolar disorder (Dhir and Kulkarni, 2011; Gulati et al., 2017; Zhou et al., 2018; Khanra et al., 2023).

### Nitric oxide in neurodegeneration

Nitric oxide is also implicated in later-life brain disorders, including many forms of neurodegeneration. NO production in both the brain and many peripheral tissues generally declines with aging (Pie et al., 2002; Zhou et al., 2002; Ashley et al., 2012; Gupta et al., 2012), and this drop-off correlates with reduced learning plasticity (Noda et al., 1997) and increased incidence of several types of dementia (Corzo et al., 2007; Zhu et al., 2021), suggesting a protective role for NO signaling under physiological conditions in aging organisms. However, as neurodegeneration sets in, NO production in the brain often ramps back up. Excessive NO signaling and resulting peroxynitrite can become a significant source of nitrosative, nitrative, and oxidative stress in neurons, leading to toxicity (Moncada and Bolaños, 2006; Cobb and Cole, 2015). In many cases, this has clear links to its physiological functions. For example, nNOS co-transmission coordinated by glutamatergic NMDA signaling that is required for many forms of learning can drive excitotoxic neuron death in excess (Dawson et al., 1991; Rameau, 2003; Parathath et al., 2007; Rameau et al., 2007), such as in uncontrolled epilepsy (Sumadewi et al., 2023) and stroke (Qin et al., 2022). In part, the bimodal effects of NO may relate to differences between its multiple mammalian synthetic enzymes, with neuronal nNOS and vascular eNOS tending to be more neuroprotective, while inflammation-associated glial and immune iNOS activity tends to be more neurotoxic (Puzzo et al., 2006; Zhu et al., 2021; Azargoonjahromi, 2023). That said, as discussed for NMDA over-activation of nNOS, segregation of function by NOS isoform cannot fully account for neuroprotective vs neurotoxic effects of NO signaling. Other contributors to this neuroprotective/neurotoxic balance remain an important topic in the field.

That said, some hints on other factors at play can be found in the realms of sleep and addiction research. Excessive NO co-transmission is also a candidate mechanism for sleep restriction induced neuron loss (Chittora et al., 2022), potentially via accumulation of stress in the autophagic recycling pathway in neurons with sustained waking (Xie et al., 2020; Bedont et al., 2021). Indeed, both behavioral hyperactivity and specifically autophagic neuron death are caused by the sleep-restricting drug cocaine via an nNOS nitrosylation-mediated co-transmission pathway (Xu et al., 2013; Guha et al., 2016). More successful mitigation of oxidative than nitrogen stress in the brain could also contribute to findings that chronic sleep loss is sufficient in and of itself to drive neuron loss in many brain regions (Owen and Veasey, 2020), despite most oxidative stress accumulating in the gut rather than the brain during chronic sleep loss (Vaccaro et al., 2020).

Thus, nNOS co-transmission is one nitrogen metabolic mechanism that may link both aging and chronic sleep loss to the etiology of neurodegenerative diseases of aging. While I will focus on Alzheimer’s disease (AD) as an exemplar here, voluminous links to both sleep and NO have been reported for many neurodegenerative diseases. Chronic sleep loss is a well-known risk factor for AD in humans, and exacerbates pathology and behavioral deficits in animal models of both amyloid (mostly early) and tau (mostly late) driven phases of AD (Owen and Veasey, 2020; Ibrahim et al., 2024a,b). Meanwhile, excessive NO co-transmission can be induced by toxic forms of both amyloid and tau, which in turn both remodels neurotransmission and amplifies nitrogen stress in ways that can feed into AD pathology (Wang et al., 2023). As discussed previously, NO synthesis is likely increased during at least some stages of chronic sleep restriction, pointing to NO as one possible mediator linking sleep disturbance to neurodegenerative pathology. On the other hand, NO co-transmission may also be beneficial in some pathological contexts, especially early in the disease course of AD, in part by promoting protective neuroplasticity via its canonical role in LTP (Azargoonjahromi, 2023). In sum, while it seems very likely that high NO signaling in late AD is net deleterious, it is less clear to what extent NO signaling in early AD is toxic vs protective. This makes it difficult to assess how NO co-transmission may link risk factors like chronic sleep loss to the origins of AD pathology, and further research in this area is warranted.

## Polyamines: agmatine, spermidine/spermine, and putrescine-derived GABA

### Synthesis, vesicular loading, and transduction of agmatine co-transmitter

Agmatine (Agm) is a polyamine primarily produced from the decarboxylation of L-arginine, putting its synthesis in competition with NO and urea production (Figure 1). Indeed, Agm is a weak antagonist of both nNOS and iNOS, exacerbating this competition (Galea et al., 1996; Feng et al., 2002).

Agmatine (Agm) is loaded into synaptic vesicles and unevenly distributed within the brain at concentrations comparable to more classical monoamine neurotransmitters such as dopamine, norepinephrine, and serotonin (Otake et al., 1998; Reis and Regunathan, 1998; Fuxe et al., 2010). Agm co-loading with Glu was subsequently observed in several brain regions, including the hippocampus and cerebral cortex, supporting Agm’s status as a co-transmitter (Seo et al., 2011; Jing et al., 2016).

Extracellular Agm binds some adrenoreceptors and imidazole receptors with fairly high affinity, and can also block NMDA cation channels (Figure 2; Reis and Regunathan, 1998; Halaris and Piletz, 2001; Uzbay, 2012). Where studies have explicitly studied Agm as a co-transmitter, most have focused on Glu/Agm co-transmission. As such, I will primarily focus on this literature in the remainder of my discussion of Agm. In other cases where Agm alone has been shown to function as a neurotransmitter, its potential co-loading with other neurotransmitters has often not been closely examined. This is a topic with considerable interest for future research.

### Agmatine co-transmission in learning and memory

The production, synaptic localization, and extracellular release of hippocampal Agm is increased by water maze learning (Liu et al., 2008; Leitch et al., 2011; Rushaidhi et al., 2013). This largely reflects increased Agm co-transmission, as there is >90% overlap in hippocampal Glu and Agm, the levels of which rise together in response to water maze learning (Seo et al., 2011). Despite this, while exogenous Agm has been reported to enhance hippocampus-dependent spatial and recognition learning (Borikar et al., 2022), this has not been consistent across studies (McKay et al., 2002; Rastegar et al., 2011; Moosavi et al., 2014), leaving the extent to which Agm co-transmission facilitates the formation and consolidation of hippocampal spatial and recognition learning unclear. That said, Agm treatment does more consistently protect such memory traces from disruption by subsequent insults, suggesting a role for Agm co-transmission in maintaining these types of learning (Moosavi et al., 2014; Prediger, 2014; Borikar et al., 2022).

Agm more clearly facilitates avoidance learning, both at the level of the hippocampus (Farokhi et al., 2022) and via an adrenoreceptor/NOS modulated transduction pathway downstream in the locus coeruleus (Shelkar et al., 2016). In the hippocampus, Agm disrupted avoidance learning at low doses and facilitated avoidance learning at high doses, with only the latter driving LTP-enhancing kinase activity and phosphorylation (Farokhi et al., 2022). This bimodal effect of Agm in avoidance learning suggests that discrepancies between studies in spatial and recognition learning may simply reflect differences in the effective Agm dosage (or perhaps Glu/Agm ratio) experienced by the hippocampus. Further, the phosphorylation mechanisms identified are consistent with convergent effects of Agm with its co-transmitter Glu on canonical LTP pathways.

### Agmatine and sleep

While Agm has been studied in the context of sleep-adjacent phenomena, such as enhanced anti-convulsant effects when Agm is co-delivered with the sleep-promoting indolamine melatonin (Moezi et al., 2011), I was unable to locate studies of Agm in the context of sleep *per se*, let alone its potential roles as a co-transmitter in this context. That said, Agm does stimulate neuron activity in the generally wake-promoting locus coeruleus by a NOS-dependent mechanism (Ruiz-Durántez et al., 2002). This is an exciting topic for future research.

### Agmatine co-transmission in addiction

Given reports of Glu/Agm co-transmission, and Agm’s dual roles as an extracellular antagonist of NMDA receptors (Figure 2) and an intracellular antagonist of NOS, it is reasonable to hypothesize that Agm may interact with the NMDA-NOS axis involved in addiction-driven “learning” experienced during withdrawal (Uzbay, 2012). Several lines of evidence support this hypothesis. Agm inhibits symptoms of withdrawal from opioids in several species (Aricioglu-Kartal and Uzbay, 1997; Aricioglu et al., 2004; Rawls et al., 2008; Su et al., 2008), and both addiction to and withdrawal from opioids are correlated with decreased Agm availability in the rat brain (Aricioglu-Kartal and Regunathan, 2002). Agm treatment also impairs adaptation of the hippocampal Glu system induced by opioids (Wang et al., 2016), suggesting that Agm/Glu co-transmission may play a role in opioid withdrawal. Similar Agm inhibition of opioid remodeling of the Glu inputs to the nucleus accumbens may also play a role in blunting opioid withdrawal (Uzbay, 2012). While not studied in as much depth, Agm has also been shown to inhibit withdrawal syndromes associated with other drugs of abuse, including alcohol (Uzbay et al., 2000; Chimthanawala et al., 2020), amphetamines (Rawls et al., 2008), cannabinoids (Rawls et al., 2008), and benzodiazepines (Rafi et al., 2021). Heavy involvement of glutamatergic systems in Agm’s beneficial effects on opioid withdrawal suggest that its co-transmission with Glu at NMDA receptors is particularly important in this context. However, further evidence is required to confirm this, particularly given Agm’s additional role as an antagonist of NOS downstream of NMDA receptors.

### Agmatine and psychiatric disorders

While Agm may exert anxiolytic effects, findings in this area are mixed (Uzbay, 2012). Stronger evidence exists for Agm’s role in the modulation of mood. Agm alleviates symptoms of depression in preclinical models, and is a promising rapid-onset anti-depressant candidate with similar potential use cases to ketamine (Camargo and Rodrigues, 2019; Valverde et al., 2021). Agm antidepressant effects have been reported to depend upon both imidazoline and GABA receptors, and NMDA-NO axis inhibition (and less consistently between studies, perhaps also serotonin and adrenergic receptors), suggesting a complex ketamine-like rapid antidepressant mechanism that may involve Agm/Glu co-transmission (Uzbay, 2012; Gawali et al., 2017; Chen et al., 2018; Weiss et al., 2018; Neis et al., 2020).

### Agmatine co-transmission and neurodegeneration

Both total Agm and vesicular Agm co-loaded with Glu changes in a regionally specific manner with age, increasing in the hippocampus and overlying temporal cortex and decreasing in the prefrontal cortex (Gupta et al., 2012; Jing et al., 2016). Curiously, Agm negatively correlated with NOS activity only in prefrontal cortex, suggesting an aging interaction between these systems only where Agm is depleted (Gupta et al., 2012). Agm is more consistently up-regulated after brain injury, whereupon it seems to serve a protective function by dampening NMDA-NO excitotoxicity, suggesting possible roles of co-transmission role with Glu, but also potentially NOS inhibition (Kuo et al., 2007; Cunha et al., 2016).

Exogenous treatment with Agm has also proven to be protective in a number of neurodegenerative contexts, including epilepsy, stroke, and age-related dementias (Xu et al., 2018). An Agm co-transmission role in dampening excitotoxicity, similar to that observed after brain injury, is likely a major player in epilepsy and stroke. How much this role vs non-transmission-related functions of Agm mediate protection in age-related dementias is less clear, but a supporting role for Agm/Glu co-transmission is consistent with Agm protection against excitotoxicity in AD models (Zhu et al., 2003).

### Synthesis and vesicular loading of spermidine and spermine co-transmitters

The polyamines putrescine (Put), spermidine (Spd), and spermine (Spm) are ubiquitously synthesized across the kingdoms of life, including in bacteria, plants, fungi, and animals (Miller-Fleming et al., 2015). In animals, these polyamines are classically synthesized in sequence from L-ornithine, produced along with urea from the breakdown of L-arginine (Figure 1; Miller-Fleming et al., 2015). Additionally, these same three polyamines may be produced in substantial amounts from Agm in the brain (Figure 1; Gilad et al., 1996; Sastre et al., 1996; Uzbay, 2012). Symbiotic gut bacteria also supplement animals’ endogenous polyamine production, especially but not exclusively via the Agm synthetic pathway, though to what extent polyamines from this source reach the brain is not entirely clear (Miller-Fleming et al., 2015).

Regardless of how they are produced, synthesis of Put, Spd, and Spm thus competes for nitrogen equivalents with not only the urea cycle (when it is active), but also with NO and Agm production for direct neurotransmission (Figure 1). Curiously, while the expression of key polyamine synthetic enzymes are primarily neuronal in the brain, the bulk of Spd and Spm in the brain is stored in astrocytes, with the notable exception of specific neuronal sub-populations such as neurosecretory cells (Laube and Veh, 1997; Bernstein and Müller, 1999; Laube et al., 2002; Krauss et al., 2006, 2007). An elaborate and well-conserved network of enzymatic and trafficking mechanisms regulate the *de novo* synthesis and appropriate localization of polyamines under physiological conditions, and in turn couple the production of other nitrergic co-transmitters to polyamine homeostasis (Figure 1; Miller-Fleming et al., 2015).

Early work showed that Put, Spd, and Spm are enriched at synaptic terminals, but concluded that these polyamines are not themselves released as neurotransmitters (Seiler and Deckardt, 1976). However, later work showed comparable membrane potential-dependent transport of Spd and Spm in glia, and similar transport more specific for Spm in neuronal synapses (Masuko et al., 2003). A vesicular polyamine transporter (VPAT) was subsequently identified that is closely related to vesicular transporters for more canonical monoamine neurotransmitters. VPAT is localized to neuronal synaptic vesicles, synaptic vesicle-like “microvesicles” in astrocytes, and mast cell secretory granules in the immune system (Hiasa et al., 2014; Takeuchi et al., 2017; Moriyama et al., 2020). VPAT is a relatively promiscuous proton antiporter that can load vesicles with Spd, Spm, and serotonin, to a lesser extent acetylcholine, and possibly also Agm and histamine (Hiasa et al., 2014). Furthermore, knockdown of VPAT reduces Spd and Spm release from astrocytes and mast cells (Hiasa et al., 2014; Takeuchi et al., 2017). It is currently unclear whether the greater Spm > Spd selectivity of neuronal synaptic vesicles suggests the existence of a distinct neuronal transporter, or the presence of one or more factors modifying VPAT selectivity in neurons (Masuko et al., 2003).

### Transduction of spermidine and spermine co-transmitters

Polyamines interact with a range of receptors and ion channels on the intracellular side of the cell membrane, conveying inward rectification in some cases and acting as modulators in others (Moriyama et al., 2020). However, Spd and Spm also have a number of extracellular neuromodulatory effects on NMDA receptors that vary with subunit composition, membrane potential, and ligand concentration, which can drive varying combinations of potentiation of the receptor, blockade of the receptor’s ion channel, and reduced affinity for Glu (Figure 2; Williams, 1997; Ogden and Traynelis, 2011). Importantly, resting extracellular polyamine concentrations are lower than needed to robustly exert NMDA modulatory effects, and NMDA receptor activation triggers extracellular Spd and Spm release in the striatum (Fage et al., 1992; Ogden and Traynelis, 2011). Thus, it is very likely that extracellular polyamine modulation of NMDA receptors reflects Spd/Spm co-neurotransmission, adding an additional layer of coincidence detection onto NMDA-mediated synaptic plasticity. This coordinated release scheme is reminiscent of neuromodulatory D-serine co-transmission at NMDA receptors, and may even suggest co-release of D-serine and polyamines from astrocytes under these conditions (with the caveat that D-serine gliotransmission is controversial) (Figure 2; Moriyama et al., 2020).

While Glu receptors generally and NMDA receptors in particular have received the lion’s share of attention for Spd and Spm extracellular signal transduction to date, other receptors likely exist, presenting an exciting area for future research.

### Spermidine and spermine co-transmission in learning and memory

The neuromodulatory role of Spd/Spm co-transmission at NMDA receptors suggests a probable role in learning and memory. Supporting this. VPAT is highly expressed in the hippocampus, and VPAT knockout mice have depleted brain Spd/Spm that correlates with notable deficits in hippocampus-dependent spatial and object recognition learning (Hiasa et al., 2014; Fredriksson et al., 2019). Local pharmacological inhibition of hippocampal polyamine synthesis caused similar impairment in object recognition, but not spatial learning (Gupta et al., 2009). However, Put levels were depleted more effectively than Spd/Spm in Gupta et al, making this weaker result consistent with Spd/Spm co-transmission (Gupta et al., 2009; Fredriksson et al., 2019). These studies highlight a common challenge when assessing the behavioral effects of polyamines; the extensive mechanisms that regulate polyamine synthesis and trafficking often complicate efforts to modify their levels, especially locally (Miller-Fleming et al., 2015).

### Spermidine and spermine in sleep, addiction, and psychiatric/neurological illness

While Spd and Spm are implicated in all of these topics, current studies were generally not designed to explicitly test the role of Spd/Spm co-transmission, making the etiology of these effects difficult to distinguish from the many critical non-signaling roles of Spd and Spm (Miller-Fleming et al., 2015). One exception is in addiction, where VPAT knockout mice have been shown to be more sensitive to amphetamine and less sensitive to diazepam, suggesting a role for Spd/Spm co-transmission (Fredriksson et al., 2019). Exogenous Spd has also been reported to alleviate many symptoms of ketamine psychosis in rodent models, which may reflect Spd co-transmission at NMDA receptors (Yadav et al., 2018). This is an area ripe for future research.

### Synthesis, vesicular loading, and transduction of putrescine-derived GABA co-transmitter

Compared to the more elaborated polyamines Spd and Spm, there is relatively little evidence that Put itself often functions as a neurotransmitter. However, the inhibitory neurotransmitter GABA can be synthesized by two distinct pathways in the brain. The canonical and most widespread pathway is via the action of glutamate decarboxylase (GAD) enzymes on Glu. But GABA can also be oxidatively synthesized from Put, by either acetylation and subsequent oxidation by monoamine oxidase (seen more often in glia), or oxidation by diamine oxidase and subsequent dehydrogenation (seen more often in neurons) (Figure 1; Kwak et al., 2020; Lim et al., 2023).

A wide range of neurons can co-release GABA, including primarily glycinergic, glutamatergic, acetylcholinergic, and dopaminergic (DA) populations, and at least some of these likely utilize Put-GABA synthesis (Tritsch et al., 2016). In addition to the canonical vesicular GABA transporter VGAT, GABA can also be loaded by vesicular transporters whose canonical substrates are other neurotransmitters, including the monoaminergic transporter VMAT2. For example, some striatal DA neurons co-load GABA via VMAT2, albeit into separate pools of DA and GABA vesicles that seem to be regulated somewhat distinctly within the same neurons (Zych and Ford, 2022).

Striatal DA/GABA neurons also do not express substantial GAD, suggesting a non-canonical pathway for acquiring GABA (Tritsch et al., 2012). I will discuss these neurons in some depth, as an exemplar for the likelihood of Put-GABA playing a role in co-transmission. In an ongoing debate, two major sources have been proposed: *de novo* synthesis of Put-GABA by an aldehyde dehydrogenase 1a1 (Aldh1a1) dependent pathway and/or reuptake of extracellular GABA originating in other cells by the transporter GAT1 (Tritsch et al., 2014; Kim et al., 2015; Melani and Tritsch, 2022; Patel et al., 2024). Genetic knockout and pharmacological blockade of both Aldh1a1 and GAT1 have been reported to reduce GABAergic inhibitory post-synaptic currents (IPSCs) in striatal DA/GABA neurons (Tritsch et al., 2014; Kim et al., 2015; Melani and Tritsch, 2022). Recently published data purporting to resolve this question shows that over-expressing GAD but not Aldh1a1 rescues GAT1-knockout DA/GABA neuron GABAergic IPSCs, disproving a role for Put-GABA *de novo* synthesis in striatal DA/GABA neurons (Melani and Tritsch, 2022). However, as discussed elsewhere in this review, competition for L-arginine and L-ornithine substrates by synthetic enzymes is often rate-limiting in the synthesis of nitrergic co-transmitters (Figure 1), and the absolute level of available Put in the brain is generally fairly low (Malik et al., 2020; Bedont et al., 2023). A limiting effect of substrate availability could easily explain increased IPSCs with GAD but not Aldh1a1 over-expression in GAT1-knockout animals (Melani and Tritsch, 2022).

Synthesizing the reports of these groups, I propose a model reminiscent of the classical treatment of DA itself. DA is both synthesized and recycled by reuptake in the striatum, and DA subjects neurons that handle it to considerable oxidative stress (Zhang et al., 2019). A similar model of both *de novo* Put-GABA synthesis and reuptake of extracellular Put-GABA (and potentially also some non-cell-autonomous GABA) via GAT1 offers considerable advantages. In this scheme, these neurons could produce their own GABA on-demand when needed, while also reducing the need for excessive Put-GABA synthesis that would exacerbate already-high oxidative stress in these neurons (Ju et al., 2022; Del Rey et al., 2024; Figure 1). Importantly, this model is consistent with requirements for both Aldh1a1 and GAT for robust striatal DA/GABA IPSCs (Tritsch et al., 2014; Kim et al., 2015; Melani and Tritsch, 2022). While some discrepancies remain, most notably opposing findings on the size of IPSC reduction after *Aldh1a1* knockout (Kim et al., 2015; Melani and Tritsch, 2022), a combined model nonetheless seems likely.

Whatever its source, one of the major circuit functions of striatal GABA co-transmission with DA appears to be binding auto-receptors to modulate phasic DA release (Patel et al., 2024). Astrocytes also synthesize and release Put-GABA, often as a source of tonic inhibition to various neural circuits (Laschet et al., 1992; Yoon et al., 2014; Kwak et al., 2020). While it is unclear to what extent this reflects cell-autonomous co-transmission, tonic glial Put-GABA release will probabilistically often non-cell-autonomously co-transmit with neuronal neurotransmitter release at the synapse. I will focus on these two relatively well-studied exemplars in exploring the functional implications of Put-GABAergic co-transmission.

### Put-GABA co-transmission in learning

While research in this area has been sporadic, early results are promising. The clearest role for Put-GABA has been shown in fear extinction memory, where distinct but complementary inhibitory functions of striatal DA/Put-GABA co-release onto intercalated cells has been described (Aksoy-Aksel et al., 2021). In the striatum, Aldh1a1(+) DA neurons are required for motor learning; however, the role of likely Put-GABA co-transmission in this process was not examined (Wu et al., 2019). Excess glial Put-GABA in both depression and AD has also been associated with impaired synaptic plasticity and/or learning (Jo et al., 2014; Srivastava et al., 2020).

### Put-GABA co-transmission in circadian rhythms and sleep

The suprachiasmatic nucleus (SCN) of the hypothalamus serves as the master light-entrained clock in mammals, exerting circadian control over sleep (Moore, 2007). As with a range of other behaviors, this regulation allows for anticipation of need at optimal times of the solar cycle conducive to sleep. In recent years, an activity pattern in astrocytes opposed to that of neurons, driven by anti-phase rhythms in Ca2 + signaling, has been shown to be a critical component of SCN function (Brancaccio et al., 2017, 2019; Patton et al., 2023). Both astrocytic uptake of neuronally derived GABA and cell-autonomous astrocytic synthesis of Put-GABA have been proposed as sources of this circadian GABA signaling (Patton et al., 2023; Ness et al., 2024). Similarly to the dueling extracellular GABA uptake vs intrinsic Put-GABA synthesis models of the source for striatal GABA released from DA neurons, these two proposed mechanisms of SCN astrocyte regulation of GABA neurotransmission are not mutually exclusive. While a definitive determination awaits future work, some manner of combined model seems likely.

In contrast, the role of Put-GABA in sleep homeostasis (i.e., detection of current sleep need) has not been directly examined to my knowledge. However, Put has a more robust sleep-promoting effect in the fruit fly model than either L-ornithine or Spd, and blocking the conversion of Put into Spd/Spm also increases sleep (Bedont et al., 2023). Chronic but not acute sleep restriction also increases polyamine accumulation, including of acetylated polyamines consistent with metabolite flow toward Put-GABA (Xu et al., 2016; Malik et al., 2020; Bedont et al., 2023; Oishi et al., 2023). Given that GABA is generally sleep-promoting (Gottesmann, 2002), this suggests a potential behavioral homeostatic feedback loop, with nitrogen stress accumulation during chronic sleep loss promoting the synthesis of Put-GABA, and thus sleep. Whether this potential mechanism reflects glial or neuronal co-transmission is presently unclear, but potential VMAT2-dependent Put-GABA synthesis and release by monoaminergic neurons under extended waking/high nitrogen conditions is a particularly exciting possibility. Many monoaminergic neuron clusters in the brainstem and posterior hypothalamus are wake-promoting, and metabolism-gated auto-inhibition of their firing by Put-GABA would be an elegant mechanism to promote sleep under conditions of high nitrogen stress.

### Put-GABA in addiction and psychiatric disease

GABAergic IPSCs in striatal DA/GABA neurons are reduced by chronic but not acute alcohol exposure, and Put-GABA reduction in *Aldh1a1*-knockout mice increases both ethanol consumption and ethanol preference in mice (Kim et al., 2015). Given the progressive nature of Put-GABA down-regulation in DA/GABA striatal neurons with chronic ethanol consumption, as well as potential diversion of these nitrogen substrates toward NO synthesis (Figure 1), it could be worthwhile to examine whether promoting polyamine synthesis in striatal DA/GABA neurons impacts ethanol withdrawal symptoms. Studies of additional drugs of abuse are also of interest.

A potential role for dysfunctional astrocytic Put-GABA tonic inhibition has also been proposed in models of both depression and post-traumatic stress (Michels et al., 2014; Srivastava et al., 2020), suggesting roles for Put-GABA co-transmission in psychiatric disorders outside of addiction.

### Put-GABA in neurodegeneration

Astrocytic release of Put-GABA provides tonic inhibition, the release of which is amplified under conditions of excess Glu excitation (Héja et al., 2009, 2012; Bell et al., 2011; Kovács et al., 2022). In primarily excitotoxic conditions such as epilepsy, Put-GABA is thus largely protective.

On the other hand, excess glial Put-GABA synthesis can provoke other types of neurodegeneration. Polyamines generally decline in the brain with normal aging, but a sharp rise in Put and acetylated polyamines readily converted into GABA is associated with AD pathology (Minois et al., 2011; Bergin et al., 2018; Vemula et al., 2019; Jimenez Gutierrez et al., 2023). Urea cycle induction and increased Put-GABA synthesis in glia is associated with AD pathology, and blockade of ornithine decarboxylase to force nitrogen stress away from oxidative Put-GABA synthesis and into the urea cycle attenuates AD-like pathology and behavioral defects in AD animal models (Bensemain et al., 2009; Jo et al., 2014; Ju et al., 2022; Bhalla and Lee, 2024). Similar toxic effects of Put-GABA synthesis may account for selective vulnerability of Aldh1a1(+) presumptive DA/GABA striatal neurons in the MPTP model of Parkinsonism (Del Rey et al., 2024). Glial Put-GABA likely also contributes, by driving excessive tonic inhibition that exacerbates Parkinsonism (Heo et al., 2020). Notably, the changes in polyamine levels seen in AD and Parkinson’s disease are reminiscent of those seen with chronic sleep loss, and represent another candidate nitrergic mechanism coupling sleep loss to neurodegeneration incidence.

## Conclusions and future directions

Taken together, physiological changes in nitrergic co-transmission are often defined by competition. NO and polyamine synthesis compete for often-limiting L-arginine and L-ornithine synthetic substrates (Figure 1), with important implications for key receptors of nitrergic neurotransmission. NMDA receptors are an exemplar of this dynamic at work, where the balance between primarily inhibitory effects of NO-mediated NMDA receptor nitration and the bimodal and highly concentration-dependent effects of extracellular polyamines at NMDA receptors put interactive guardrails around the range of permitted NMDA calcium flux (Figure 2; Gupta et al., 2009; Fredriksson et al., 2019; Farokhi et al., 2022; Sharma et al., 2023). These guardrails will then shift along with the composition of nitrergic co-transmitters present. Similar nitrergic interactions likely extend to other receptors that are simply not as exhaustively studied as NMDA receptors.

Thus, competition for L-arginine and related synthetic substrates likely affects not only the magnitude but also the polarity of nitrergic co-transmission on plasticity, including but not necessarily exclusively at NMDA receptors. This balance can become skewed and contribute to maladaptive plasticity, leading to aberrant “learning” underlying withdrawal syndromes and certain symptoms of non-addiction psychiatric disorders (Tayfun Uzbay and Oglesby, 2001; Peregud et al., 2006, 2008; 2016; Michels et al., 2014; Kim et al., 2015; Fredriksson et al., 2019; Srivastava et al., 2020; Sadeghi et al., 2022). Such imbalances may also contribute to other pathological states, including the early stages of neurodegeneration.

On the other hand, under conditions of high nitrogen availability, multiple nitrergic co-transmission pathways can become heavily induced in tandem. During chronic sleep restriction, high nitrogen stress leads to induction of both NO and polyamine synthesis that likely serve together to promote sleep, helping to behaviorally reduce nitrogen stress and head off neurotoxicity (Gautier-Sauvigné et al., 2005; Cespuglio et al., 2012; Xu et al., 2016; Malik et al., 2020; Chittora et al., 2022; Bedont et al., 2023; Oishi et al., 2023). On the other hand, nitrogen stress cannot always be extinguished by behavioral modification, as in the case of AD and other neurodegenerative syndromes. In these cases, sustained high nitrogen stress and resulting NO and polyamine up-regulation have mostly toxic effects, exacerbating both pathology and symptoms of the disease state (Bensemain et al., 2009; Ju et al., 2022; Wang et al., 2023; Bhalla and Lee, 2024; Del Rey et al., 2024).

Taken together, the available evidence not only demonstrates important physiological and pathological roles for nitrergic co-transmission, but makes clear that these effects are grounded in fundamental ways in both underlying constitutive differences and current state-dependent variations in brain nitrogen load. That said, much remains unknown. Most current studies utilizing pharmacological or genetic nitrergic manipulations ascribe resulting physiological and behavioral effects specifically to that system. However, these manipulations will often affect multiple nitrergic co-transmission systems, due to the interconnected synthesis and regulation of nitrergic metabolic pathways (Figure 1). The potential for “off-target” effects on nitrogen metabolism in such studies, which could impact experimental readouts, is considerable. The current gold-standard approach to assess metabolite interactions in such cases is isotopic labeling, which allows the tracking of one or more heavy elemental isotopes through the metabolome with mass spectrometry, nuclear-magnetic resonance, or similar metabolomic techniques (Wang et al., 2024). Heavy isotopes introduced in as L-arginine, L-ornithine, or other carbon/nitrogen donors to nitrergic metabolic pathways could in principle allow both on-target and off-target effects on nitrogen waste metabolism to be directly measured. This methodology is particularly promising in the context of put-GABA, as it should allow the differentiation of polyamine- vs Glu-derived pools of GABA using retention of heavy isotopes. Alternatively, when isotopic labeling is infeasible, unlabeled metabolomics and/or targeted biochemical assays can detect correlative changes in total off-target metabolite levels, while epistasis studies with multiple genetic and/or pharmacological manipulations can allow causal assessment of whether probable metabolic off-targets are required for given physiological or behavioral readouts. Where possible, future studies should endeavor to rule out off-target metabolic effects, either by confirming minimal changes in the synthesis of metabolic off-targets or demonstrating that linked nitrogen metabolism pathways are not required for observed effects.

A related difficulty is that much of the current literature does not delineate between co-neurotransmission vs more generally metabolic functions of targeted nitrergic pathways. Classical EM based approaches demonstrating changes in the vesicular co-loading of nitrogen metabolites (or for NO, changes in nitrosylation or nitration) can establish suggestive correlative evidence of co-neurotransmission effects. However, truly establishing causality often requires newer techniques. In some cases, genetic or pharmacological inhibition of key proteins required selectively for co-neurotransmission effects of nitrogen metabolites offer inroads. VPAT is particularly promising in this vein for the study of Spd/Spm co-transmission effects, albeit primarily in situations where monoaminergic neurotransmitters are not major players (Hiasa et al., 2014). In other cases, such as NO, dual manipulations of nitrergic neurotransmission systems and candidate receptors in distinct cell populations may be necessary to causally establish co-transmission roles, due to the likelihood of cell-autonomous signaling.

All told, future studies weaving together approaches old and new will be necessary to better understand how nitrogen metabolism drives whole-organism physiology and behavior. Future work should expand our understanding of how individual nitrergic systems such as NO, polyamine, and polyamine derivatives such as put-GABA contribute to both physiological and pathological whole-organism state and behavior. But beyond this, forward-thinking work will ideally also improve comprehension of how the integrated network of nitrogen metabolism may contribute to such effects by sometimes-unexpected indirect mechanisms, and better distinguish between co-transmission and cell-autonomous metabolic effects. Multimodal approaches will be necessary to definitively answer the central question of this review: how does crosstalk across the metabolic, physiological, and behavioral levels ultimately link nitrogen load to goal-directed behavior?
